# Fish Oil Supplementation Alters the Plasma Lipidomic Profile and Increases Long-Chain PUFAs of Phospholipids and Triglycerides in Healthy Subjects

**DOI:** 10.1371/journal.pone.0042550

**Published:** 2012-08-28

**Authors:** Inger Ottestad, Sahar Hassani, Grethe I. Borge, Achim Kohler, Gjermund Vogt, Tuulia Hyötyläinen, Matej Orešič, Kirsti W. Brønner, Kirsten B. Holven, Stine M. Ulven, Mari C. W. Myhrstad

**Affiliations:** 1 Department of Health, Nutrition and Management, Faculty of Health Sciences, Oslo and Akershus University College of Applied Sciences, Oslo, Norway; 2 Department of Nutrition, Institute for Basic Medical Sciences, University of Oslo, Oslo, Norway; 3 Nofima, Norwegian Institute of Food, Fisheries and Aquaculture Research, Ås, Norway; 4 Centre for Integrative Genetics (CIGENE), Department of Mathematical Sciences and Technology, Norwegian University of Life Science, Ås, Norway; 5 VTT Technical Research Centre of Finland, Espoo, Finland; 6 TINE SA, Centre for Research and Development, Kalbakken, Oslo, Norway; Institut Pluridisciplinaire Hubert Curien, France

## Abstract

**Background:**

While beneficial health effects of fish and fish oil consumption are well documented, the incorporation of n-3 polyunsaturated fatty acids in plasma lipid classes is not completely understood. The aim of this study was to investigate the effect of fish oil supplementation on the plasma lipidomic profile in healthy subjects.

**Methodology/Principal Findings:**

In a double-blinded randomized controlled parallel-group study, healthy subjects received capsules containing either 8 g/d of fish oil (FO) (1.6 g/d EPA+DHA) (n = 16) or 8 g/d of high oleic sunflower oil (HOSO) (n = 17) for seven weeks. During the first three weeks of intervention, the subjects completed a fully controlled diet period. BMI and total serum triglycerides, total-, LDL- and HDL-cholesterol were unchanged during the intervention period. Lipidomic analyses were performed using Ultra Performance Liquid Chromatography (UPLC) coupled to electrospray ionization quadrupole time-of-flight mass spectrometry (QTOFMS), where 568 lipids were detected and 260 identified. Both t-tests and Multi-Block Partial Least Square Regression (MBPLSR) analysis were performed for analysing differences between the intervention groups. The intervention groups were well separated by the lipidomic data after three weeks of intervention. Several lipid classes such as phosphatidylcholine, phosphatidylethanolamine, lysophosphatidylcholine, sphingomyelin, phosphatidylserine, phosphatidylglycerol, and triglycerides contributed strongly to this separation. Twenty-three lipids were significantly decreased (FDR<0.05) in the FO group after three weeks compared with the HOSO group, whereas fifty-one were increased including selected phospholipids and triglycerides of long-chain polyunsaturated fatty acids. After seven weeks of intervention the two intervention groups showed similar grouping.

**Conclusions/Significance:**

In healthy subjects, fish oil supplementation alters lipid metabolism and increases the proportion of phospholipids and triglycerides containing long-chain polyunsaturated fatty acids. Whether the beneficial effects of fish oil supplementation may be explained by a remodeling of the plasma lipids into phospholipids and triglycerides of long-chain polyunsaturated fatty acids needs to be further investigated.

**Trial Registration:**

ClinicalTrials.gov NCT01034423

## Introduction

Intake of fish and fish oil, containing n-3 fatty acids; eicosapentaenoic acid (EPA; 20∶5) and docosahexaenoic acid (DHA; 22∶6), is associated with beneficial health effects such as reduced risk of cardiovascular disease and sudden cardiac death [1]–[4]. The beneficial effects of marine n-3 fatty acids have been explained by decreased plasma triglycerides (TGs) [5], [6], moderate reduction in blood pressure [7], reduced platelet aggregation [8], [9], and protection against cardiac arrhythmias [10], [11]. It has been suggested that bioactive lipid components may be important in mediating these effects, but the molecular mechanisms are still to a large extent unknown.

Cells, tissues and biological fluids contain tens of thousands of structurally different lipids, that fulfil multiple roles in cellular signalling, in membrane structure, and as fuel sources for many cell types [12]. The entire spectrum of lipids in a biological system, can be defined as the lipidome [13], which combines mass spectrometry technology and bioinformatics methods with traditional methods such as sample preparation, lipid extraction and separation. Lipidome analyses have revealed a diversity of lipid compounds in human plasma, which can be classified into six main lipid categories including fatty acyls, glycerolipids, glycerophospholipids, sphingolipids, sterol lipids and prenol lipids [14]. The major plasma lipids are the glycerolipids (TGs), glycerophospholipids (phospholipids) and sterol lipids which are transported in the lipoprotein particles [14], [15].

In n-3 FA intervention studies fatty acids have been measured in different blood compartments such as in platelets and red blood cells, and in plasma cholesteryl esters, triglycerides and phospholipids. Lipidomic analysis now offers the opportunity to detect exact fatty acid composition of these individual lipids. [16]. Recently it was shown that the plasma lipidomic profile was altered in subjects with coronary heart disease after intake of fatty fish, and in subjects with metabolic syndrome after consumption of a healthy diet containing fatty fish, wholegrain products and bilberries [17], [18]. Furthermore, profiling of the plasma lipids suggests a relationship between the composition of plasma lipids and diet [19], [20], with diet-induced weight loss [21] and diet-related diseases such as diabetes mellitus [22]. This opens up the opportunity to identify new functional lipid biomarkers to detect and prevent diet-related diseases. We have however not been able to find studies showing the plasma lipidomic profile in healthy subjects after intake of fish oil.

We have previously reported that in the present study a daily intake of fish oil (1.6 g EPA+DHA/d) did not change the level of serum lipids, markers of oxidative stress, lipid oxidation or inflammation, whereas an increase in plasma EPA, DPA and DHA was observed after three and seven weeks of intervention in a randomized controlled study in healthy subjects [23]. The aim of this study was to apply a lipidomic strategy to further describe the effect of fish oil supplementation in healthy subjects.

## Materials and Methods

### Subjects

Healthy men and women between 18–50 years were recruited into this study. Detailed description of the protocol, participant recruitment and enrolment, inclusion and exclusion criteria, and compliance are described in details elsewhere [23]. In brief, exclusion criteria were total cholesterol >7.5 mmol/l, triglycerides >4 mmol/l, glucose >6.0 mmol/l, C-reactive protein (CRP) >10 mg/l, body mass index (BMI) ≥30 kg/m^2^ and blood pressure (≥160/100). The study was performed at the Akershus University College, Norway between September and December 2009.

### Ethics Statement

Written informed consent was obtained from all participants and the protocol was approved by the Regional Committee of Medical Ethics (approval no. 6.2008.2215) and by the Norwegian Social Science Data Services (approval no. 21924), and was conducted in accordance with the Declaration of Helsinki.

### Study Design

**Figure 1:**
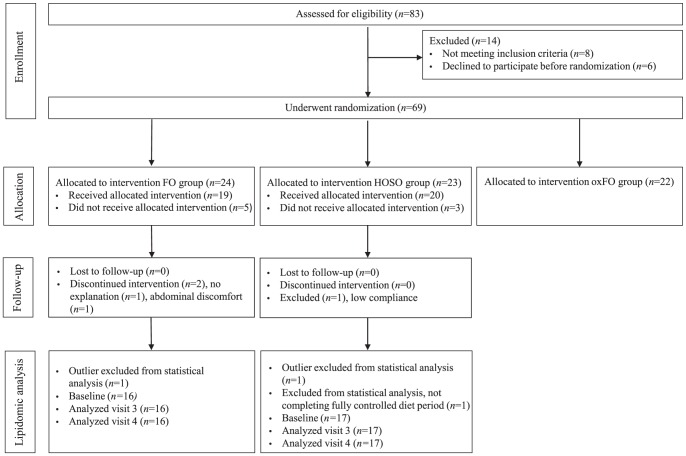
Flow chart of the study showing subjects enrolled, lost during follow-up and number of subjects included in the statistical analysis at baseline and after three and seven weeks of fish oil supplementation. FO group, fish oil group; HOSO, high oleic sunflower oil group; oxFO, oxidized fish oil group (not included in the present study).

This study was a part of a randomized controlled double-blinded three-arm parallel group study, designed to investigate health effects from intake of fish oil [23]. In the present study, data from two of the intervention groups are included, as shown in Figure 1. Subjects in the present study were given 8 g/d of either fish oil (FO) or high oleic sunflower oil (HOSO), and each subject was taking 16 capsules/d minimum twice each day for seven weeks. Subjects in the fish oil group received capsules containing 0.7 g/d EPA+0.9 g/d DHA from cod liver oil (Gadidae sp., TINE EPADHA Oil 1200) provided by TINE SA (Oslo, Norway) and subjects in the control group received high oleic sunflower oil purchased from AarhusKarlshamn AB (Malmø, Sweden). The subjects were instructed to take the capsules with food (minimum two meals). The fatty acid composition in the oils has been described elsewhere [23].

The subjects met for visits and blood samples for the lipidome analyses were collected at 0, 3 and 7 weeks. Between the screening and baseline visit (week 0), the subjects conducted a four-week washout period, where foods containing marine n-3 fatty acids were avoided. During the first three weeks of the intervention the subjects conducted a fully-controlled isocaloric diet, provided with all food and beverages at Akershus University College, Norway. The mean daily intake during the fully controlled diet period (exclusive capsules, n = 33) was 9.1±2.3 MJ and the composition of the diet consumed was similar in both groups containing 24.4±1.1 percentage of energy (E%) from fat, of which 7.8±0.3 E% from SFA, 5.8±0.4 E% from MUFA and 4.8±0.9% PUFA. The carbohydrate content was 54.4±1.1 E%, including 4.6±0.9 E% from added sugar and 19.4±0.5 E% from protein. The food items provided in this study have previously been described [23]. The last four weeks of the intervention period the subjects returned to their habitual diet. Intake of fish, fish products, marine n-3 enriched food or dietary supplements was not allowed during the entire study period of 11 weeks. The study was registered at www.clinicaltrial.gov (IDno. NCT01034423).

### Blood sampling

Subjects were told to refrain from alcohol consumption and vigorous physical activity the day prior to blood sampling. Venous blood samples were drawn after an overnight fast (≥12 hours) at the same time (±2 h) and serum were kept at room temperature at 30 min before centrifuged (1500 *g* 12 min). EDTA-plasma was immediately placed on ice and centrifuged within 10 min (1500 *g*, 4°C, 10 min). N_2_ flushed plasma samples were snap frozen and stored at −80°C until further analysis.

### Routine laboratory analysis

Fasting serum hsCRP, total- cholesterol, LDL-cholesterol, HDL-cholesterol, triglycerides and glucose were measured by standard methods at a routine laboratory (Fürst Medical Laboratory, Oslo, Norway).

### Lipidomic analyses

An aliquot (10 µL) of plasma sample was diluted with 10 µL of 0.15 M (0.9%) sodium chloride and 10 µL of internal standard mixture containing PC(17:0/0:0), PC(17:0/17:0), PE(17:0/17:0), PG(17:0/17:0)[rac], Cer(d18:1/17:0), PS(17:0/17:0) and PA(17:0/17:0) (Avanti Polar Lipids, Inc., Alabaster, AL, USA) and TG(17:0/17:0/17:0) and MG(17:0/0:0/0:0)[rac], DG(17:0/17:0/0:0)[rac] (Larodan Fine Chemicals) was added. The lipids were extracted using a mixture of HPLC-grade chloroform and methanol (2∶1; 100 µL). The lower phase was collected (60 µL) and 10 µL internal standard mixture containing labeled PC(16:1/0:0-D_3_), PC(16:1/16:1-D_6_) and TG(16:0/16:0/16:0-^13^C3) was added.

The extracts were analyzed on a Waters Q-Tof Premier mass spectrometer combined with an Acquity Ultra Performance LC™ (UPLC) in randomized order. The column (at 50°C) was an Acquity UPLC™ BEH C18 2.1×100 mm with 1.7 µm particles. The solvent system included A: ultrapure water with 1% 1 M NH_4_Ac and 0.1% HCOOH, and B: LC/MS grade acetonitrile/isopropanol (1∶1) with 1% 1M NH_4_Ac and 0.1% HCOOH. The gradient started from 65% A/35% B, reached 80% B in 2 min, 100% B in 7 min and remained there for 7 min. The flow rate was 0.400 ml/min and the injected amount was 2.0 µl (Acquity Sample Organizer, at 10°C). Reserpine was used as the lock spray reference compound. The lipid profiling was carried out using ESI+ mode and the data was collected at mass range of m/z 300–1200 with scan duration of 0.2 sec. The data was processed by using MZmine2 software [24] and the lipid identification was based on an internal spectral library.

The data processing included peak detection, integration, peak alignment, normalization and identification. Lipids were identified using an internal spectral library. The data was normalized using internal standards representative of each class of lipid present in the samples: the intensity of each identified lipid was normalized by dividing it with the intensity of its corresponding standard and multiplying it by the concentration of the standard. All monoacyl lipids except cholesterol esters, such as monoacylglycerols and monoacylglycerophospholipids, are normalized with PC(17:0/0:0), all diacyl lipids except ethanolamine phospholipids are normalized with PC(17:0/17:0), all ceramides with Cer(d18:1/17:0), all diacyl ethanolamine phospholipids with PE(17:0/17:0), and TG and cholesterol esters with TG(17:0/17:0/17:0).

### Statistical analyses

Sample size was calculated using expected change in plasma n-3 fatty acids as described as previously described [23]. Multi-Block Partial Least Squares Regression (MBPLSR) analysis was used for exploring the sample and variable variation patterns in the data [25] where each lipid class was defined as one individual block [26] resulting in 11 blocks of descriptor variables in total (i.e. 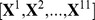). The multi-block set of descriptor variables were organized in the following order: Ceramides as block one (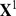), lysophosphatidylcholines (lysoPC) as block two (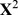), lysophosphatidylethanolamines (lysoPE) as block three (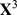), phosphatidic acid (PA) as block four (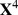), phosphatidylcholines (PC) as block five (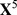), phosphatidylethanolamines (PE) as block six (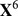), phosphatidylglycerols (PG) as block seven (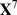), phosphatidylserines (PS) as block eight (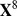), sphingomyelins (SM) as block nine (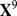), triglycerides (TG) as block ten (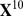) and sums of lipid classes together with phosphatidylinositol (PI) as block eleven (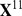) (a separate block was not assigned to PI class since it contained only one lipid). An intervention group indicator variable was used as response variable (*y*-variable). In order to estimate both the influence of the total amount of lipids in each lipid class and simultaneously the influence of the relative variation within each lipid class, each block was normalized by a division by the total amount of lipids in the corresponding class. The total amounts of lipids of each lipid classes were then used as an additional block and named “the sums of lipids”. Subsequently, plasma lipids were transformed by taking the log2 ratio (baseline adjusted log2 values) after three and seven weeks in both the FO and the HOSO group. After this, each block (lipid class) was set on the same footing prior to MBPLSR analysis by block scaling as described in [26]. Model-validation and testing of the influence of each lipid class to the global model was done by cross-validation as described in [26].Variable significance testing for the difference between the groups at baseline and after the intervention (baseline adjusted values) was done by cross-validation [25], [26] of the multivariate MBPLSR model and by univariate testing using Student's- *t* test. For the univariate testing the log2 ratios were used and False Discovery Rate (FDR) corrected *q*-values were computed using the R package ‘qvalue’. Two subjects were detected as outliers by the MBPLSR models and were therefore excluded from the further analysis. Baseline characteristics were analyzed using (baseline adjusted values) Student's- *t* test and Mann Whitney U test (serum triglycerides) when data was normally and not normally distributed, respectively. The significance level was set to 5% (two-sided) and the power of the test was chosen to be 80%. Data in Table 1 are presented as mean ± SD. All univariate analyses were performed using SPSS for windows (SPSS, version 19.0) and multivariate analyses were performed using in-house-written and standard MATLAB routines (MATLAB version 7.8).

**Table 1:** BMI and serum lipids at baseline and after three weeks of intervention with fish oil (n = 16) and high oleic sunflower oil (n = 17).

	Fish oil		Sunflower oil			
Parameter	Baseline	3 wk	Baseline	3 wk	P-value*	P-value**
BMI (kg/m^2^)	22±3	22±3	23±3	23±3	0.25	0.53
Triglycerides (mmol/l)	0.9±0.4	0.9±0.3	1.1±0.7	1.1±0.4	0.46	0.68
Total-cholesterol (mmol/l)	4.6±0.8	4.4±0.6	4.9±0.9	4.6±1.0	0.27	0.36
LDL-cholesterol (mmol/l)	2.5±0.8	2.4±0.8	2.7±0.6	2.6±0.6	0.35	0.64
HDL-cholesterol (mmol/l)	1.5±0.3	1.4±0.4	1.5±0.4	1.4±0.4	1	0.86

* Independent t- test for between groups at baseline.

** Independent t- test for changes between groups after three weeks.

## Results

### Characteristics of the subjects

A total of 33 normal weight healthy subjects (n = 8 men and n = 25 women) completed this study. The subjects were young (28±8 years), and with serum lipids within the normal range as shown in Table 1. No differences in age, BMI or serum lipids were observed between the FO group (n = 16) and the HOSO group (n = 17) at baseline (Table 1). Serum lipids and BMI were not significantly changed between the groups after three (Table 1) or seven weeks of intervention (data not shown).

### Plasma lipidomic profile

A total of 568 lipids were detected and quantified in the plasma samples of the two intervention groups. Of these lipids, 260 were identified, including the following lipid classes; ceramides, sphingomyelins (SM), lysophosphatidylcholines (lysoPC), lysophosphatidylethanolamines (lysoPE), phosphatidic acids (PA), phosphatidylcholines (PC), phosphatidyethanolamines (PE), phosphatidylglycerols (PG), phosphatidylinositols (PI), phosphatidyserines (PS) and triglycerides (TG). In the present study, the identified lipids were included in the statistical analysis.

**Figure 2:**
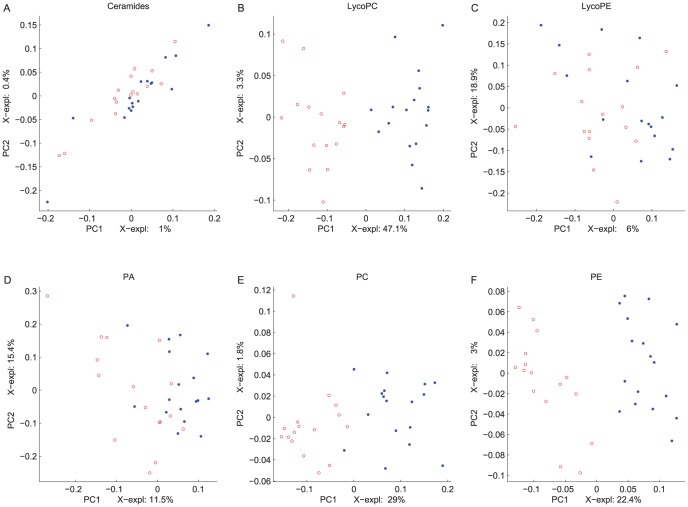
Multi-Block Partial Least Squares Regression (MBPLSR) analysis of the data after three weeks of intervention. First and second PLSR components of block scores of ceramides, lysoPC, lysoPE, PA, PC and PE are shown (A–F). The samples of each intervention group are presented as blue (HOSO group) or red (FO group) circles. The (un-validated) explained variances are shown on the axes.

**Figure 3:**
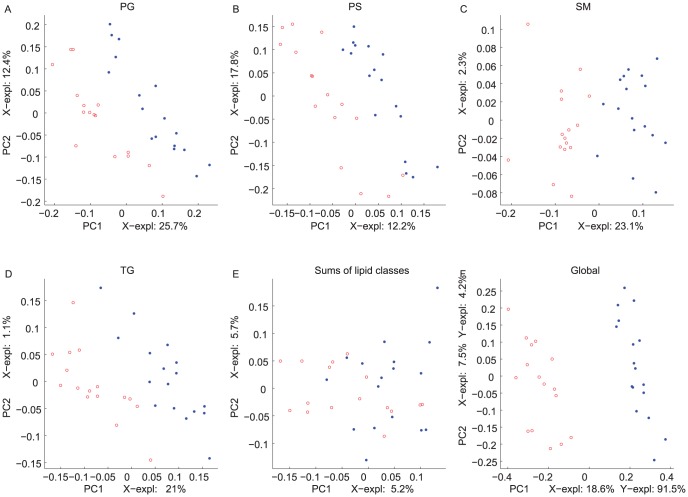
Multi-Block Partial Least Squares Regression (MBPLSR) analysis of the data after three weeks of intervention. First and second PLSR components of block scores of PG, PS, SM, TG, the sums of lipid classes and global scores are shown (A–F). The samples of each intervention group are presented as blue (HOSO group) or red (FO group) circles. The (un-validated) explained variances are shown on the axes.

**Figure 4:**
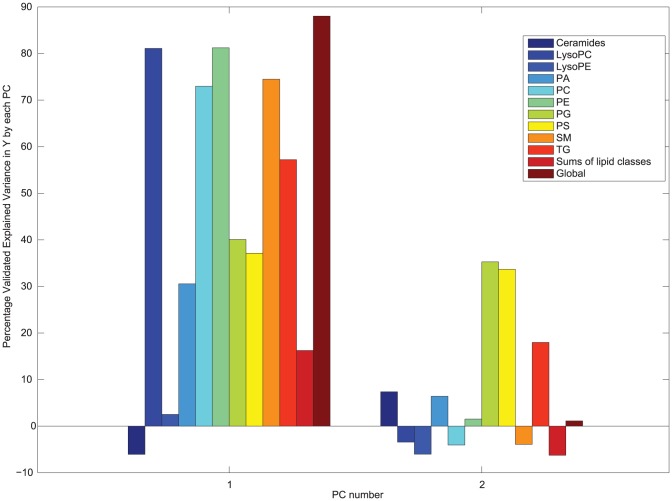
Cross-validated explained variance in Y. Bar plots of the validated explained variances in **Y** for each block and for the global model using data obtained after three weeks of intervention are presented.

MBPLSR was performed with lipid class blocks as a multi-block **X** and intervention group indicator variable as y-variable. The lipid class block variation patterns after three weeks of intervention are shown in Figure 2 and Figure 3A–D. The lipidomic profiles of the two intervention groups were well separated in the global sample variation pattern (global score plot) (Figure 3F). The first principal component accounted for most of the separation of the two groups and explained 91.5% of the total variance in the y-variable. The explained block variances are shown on the respective axes. Several lipid class blocks (lysoPC, PC, PE, PG, PS, SM and TG) showed a clear separation of the FO and the HOSO group, whereas ceramides, lysoPE and PA lipids did not separate the FO and the HOSO group (Figure 2 and Figure 3A–D). In addition, the sums of lipid classes did not separate the FO and the HOSO group (Figure 3E) showing that the differences between FO and HOSO group can be explained by remodelling within lipid classes rather than by changes in the total amount of lipids in each class. A similar separation of the groups and patterns in block score plots were observed also after seven weeks of intervention (data not shown). To further analyze the plasma lipid profile and to identify specific lipids contributing to the separation, we decided to use the data obtained after completing a three weeks fully controlled diet period. After three weeks of intervention, the contribution of each lipid class block to the prediction of the group indicator variable was validated by calculating root mean squared errors of cross-validation per block. The validated explained variances for the first two components are shown in Figure 4. The first principal component of the lysoPC, PC, PE and SM lipid classes described most of the group separation and explained more than 70% of the y-variance. The second component of the PG, PS and TG lipids explained the separation of the two intervention groups further accounting for 19–37% of the y-variance. By significance testing using cross-validation and Jack-knifing [26] a total of 75 lipids were identified as significant for the separation of the two intervention groups after three weeks using a two principal component model (Table S1). By investigating the validated root mean squared error as a function of components a two component model was selected (Figure S1).

**Figure 5:**
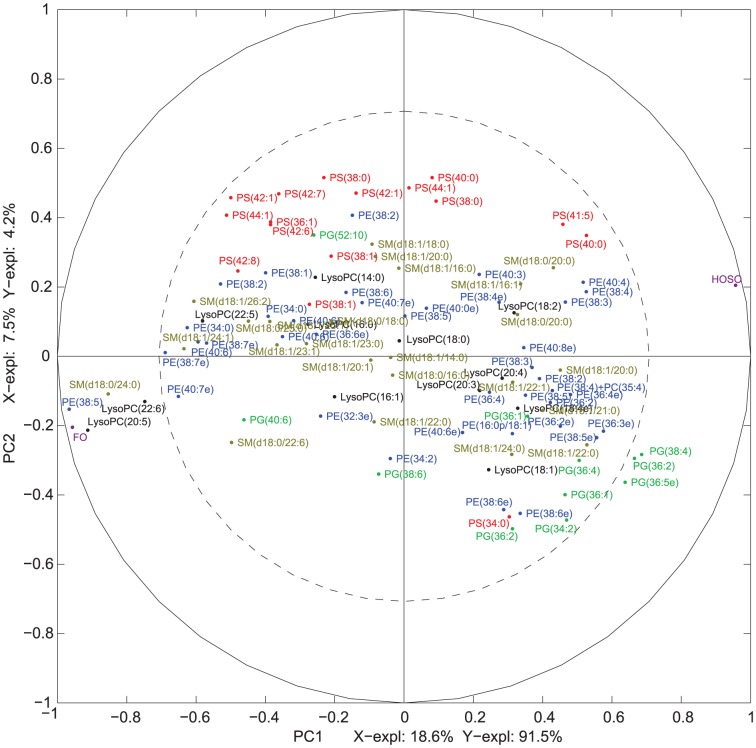
Multi-Block Partial Least Squares Regression (MBPLSR) analysis of the data after three weeks of intervention. Correlation loading plot for the variables contributing to the separation of the FO and the HOSO group after three weeks are shown for LycoPC, PE, PG, PS and SM. The (un-validated) explained variances in **X** and **Y** are shown on the axes.

**Figure 6:**
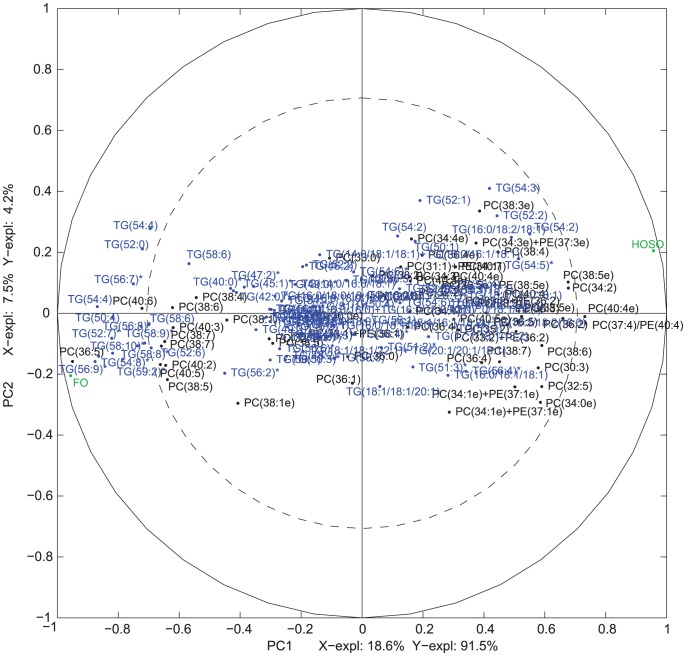
Multi-Block Partial Least Squares Regression (MBPLSR) analysis of the data after three weeks of intervention. Correlation loading plot for the variables contributing to the separation of the FO and the HOSO group after three weeks are shown for TG and PC. The (un-validated) explained variances in **X** and **Y** are shown on the axes.

To further identify the specific lipids that contributed to the distinction of the FO and the HOSO group correlation loading plots were studied [26]. The correlation loading plots in Figures 5 and 6 show that several phospholipids and TGs containing long-chain PUFAs including lysoPC(20:5), lysoPC(22:6), PC(36:5), PC(40:6), PE(38:5), TG(50:4), TG(52:6), TG(52:7), TG(54:8), TG(56:7), TG(56:8), TG(56:9), TG(58:6), TG(58:8), TG(58:9), and TG(58:10) were strongly positively correlated to intake of FO supplementation.

A strong positive correlation was also observed between intake of FO supplementation and lipids of long-chain and lower double bond content such as SM(18:0/24:0), TG(59:2) and TG(52:0). Only PC(40:4e), PC(37:4)/PE(40:4), PC(38:5) and PC(34:2) were found negatively correlated to the FO group.

In order to describe altered lipids in the FO group compared to the HOSO group unpaired t-test was performed. In the FO group, 74 lipids were significantly altered (FDR<0.05) compared with the HOSO group after three weeks of intervention, and 51 out of these 74 lipids were significantly increased. Several phospholipids and TGs containing long-chain PUFAs were increased in the FO group, compared to the HOSO group. Significantly altered lysoPC, PC, PE, PA, PG, PS, PI, SM and TG lipids are shown in Tables 2 and 3. Furthermore, 49 lipids were identified as significantly altered in the FO group compared to the HOSO group by both unpaired t-test and MBPLSR (Table S1). After seven weeks of intervention 58 significant altered lipids were identified in the FO group compared to the HOSO group, and 33 out of these 58 lipids were significantly altered after both three and seven weeks (data not shown).

**Table 2:** Significantly altered lipids (FDR<0.05) in the fish oil (FO) group compared to the sunflower oil group (HOSO) after three weeks of intervention.

		Fold change from baseline	
Lipid Name	q-value	HOSO	FO
LysoPC(20:5)	<0.001	0.80	4.35
LysoPC(22:5)	0.025	0.91	1.67
LysoPC(22:6)	0.003	0.94	1.89
PA(38:5e)	0.006	1.05	0.75
PE(38:4)	0.026	1.11	0.88
PE(38:4)+PC(35:4)	0.029	1.27	0.90
PE(38:5)	<0.001	0.92	3.29
PE(38:5e)	0.042	1.09	0.75
PE(38:7e)	<0.001	1.17	2.58
PE(38:7e)	0.013	1.11	1.40
PE(40:4)	0.027	1.10	0.78
PE(40:6)	0.013	1.10	1.75
PE(40:7e)	0.010	1.08	1.51
PG(36:2)	0.015	0.96	0.68
PG(36:5e)	0.028	1.02	0.80
PG(38:4)	0.013	1.24	0.89
PG(40:6)	0.003	1.11	1.91
PI(40:7)	0.024	1.25	0.96
PS(36:1)	0.001	1.14	1.84
PS(38:0)	0.031	1.18	1.67
PS(38:1)	0.010	1.11	1.58
PS(38:1)	0.019	1.26	1.83
PS(41:5)	0.031	1.17	0.96
PS(42:1)	0.003	1.09	1.86
PS(42:6)	0.001	0.96	1.54
PS(42:7)	0.001	0.92	1.39
PS(42:8)	0.001	1.36	2.51
PS(44:1)	0.001	1.08	1.86
SM(d18:0/20:0)	0.029	1.08	0.81
SM(d18:0/22:6)	0.015	1.01	1.44
SM(d18:0/24:0)	<0.001	0.93	2.62
SM(d18:1/26:2)	0.015	1.10	1.43

**Table 3:** Significantly altered lipids (FDR<0.05) in the fish oil (FO) group compared to the sunflower oil (HOSO) group after three weeks of intervention.

		Fold change from baseline	
Lipid Name	q-value	HOSO	FO
PC(30:3)	0.015	0.85	0.6
PC(32:5)	0.024	1.03	0.77
PC(36:3)	0.031	0.97	0.73
PC(36:5)	<0.001	0.80	4.00
PC(37:4)/PE(40:4)	0.021	1.07	0.83
PC(38:1)	0.025	1.05	1.60
PC(38:1e)	0.026	0.91	1.39
PC(38:4)	0.007	2.35	9.08
PC(38:5)	<0.001	1.12	3.97
PC(38:5e)	0.006	1.2	0.96
PC(38:6)	0.006	1.15	1.52
PC(38:6)	0.037	0.89	0.69
PC(38:7)	0.001	1.09	2.27
PC(38:7)	0.001	1.09	2.25
PC(40:2)	<0.001	0.99	3.54
PC(40:3)	0.001	0.93	1.74
PC(40:4)	0.029	1.09	0.79
PC(40:4e)	0.015	1.01	0.78
PC(40:5)	<0.001	0.89	1.57
PC(40:6)	<0.001	1.04	1.71
TG(50:4)	<0.001	1.04	3.57
TG(52:0)	0.001	1.03	2.52
TG(52:2)	0.029	1.09	0.79
TG(52:6)	0.004	0.96	2.86
TG(52:7)	<0.001	0.80	4.55
TG(54:2)	0.001	1.27	0.59
TG(54:3)	0.029	1.26	0.83
TG(54:4)	<0.001	0.96	2.34
TG(54:4)	0.003	0.97	1.73
TG(54:5)	0.022	1.51	0.82
TG(54:8)	<0.001	0.98	5.02
TG(56:2)	0.026	1.02	1.93
TG(56:4)	0.038	4.50	1.47
TG(56:7)	<0.001	1.09	3.02
TG(56:8)	<0.001	1.27	2.71
TG(56:9)	<0.001	1.01	4.76
TG(58:10)	<0.001	1.17	4.14
TG(58:6)	0.003	1.17	2.10
TG(58:6)	0.023	0.99	1.70
TG(58:8)	<0.001	1.36	4.07
TG(58:9)	0.001	1.48	4.05
TG(59:2)	<0.001	1.08	2.73

## Discussion

We have investigated the effect of fish oil supplementation on the plasma lipidomic profile in healthy subjects. A clear distinction of the lipidomic profile was obtained between the FO and the HOSO group after three and seven weeks of intervention. The lipid classes that contributed to the separation of the intervention groups were LysoPC, PC, PE, PG, PS, SM and TG. FO supplementation especially increased phospholipids and TGs of long-chain PUFAs, but the total concentration of the lipids within each lipid class remained unchanged and did not differ in the FO compared to the HOSO group. The clear distinction between the FO and the HOSO group was observed after a fully-controlled isocaloric diet period for three weeks and it was also evident after the subjects had continued on their habitual diet for additionally four weeks. HOSO was used as control oil due to a similar lipid composition as the background diet, and the expected change in plasma lipidome after intake of HOSO were thought to be small.

By using MBPLSR, co-variation patterns in sample and variable space for the different lipid classes was studied. MBPLSR is a method based on latent variables, where by using only few latent variables the problem of over-fitting and false discovery is minimized. For MBPLSR analysis data blocks were organized and normalized, such that remodelling effects in each lipid class and changes in total amounts of lipids per class could be studied separately. A possible consequence of organizing the data into lipid classes is that patterns in the dataset related to other features maybe overlooked. We also included univariate analyses were the data was not organized into lipid classes. The univariate analyses will produce many false significant lipids due to the high number of variables and therefore false discovery adjusted q-values were computed. The majority of the significant altered lipids identified with univariate analyses were also identified as changed with multivariate analyses, indicating that the organization into lipid classes did not influenced the identification of significant altered lipids.

Recent results from a dietary intervention study have shown that fish intake increased TGs of long-chain PUFA similar to our results, and that fish consumption for eight weeks increased plasma long-chain TGs in subjects with coronary heart disease [18]. Interestingly, this effect was significant after intake of lean fish and not fatty fish [18]. A healthy diet rich in whole grain products, fish and bilberries significantly changed multiple TGs incorporating long-chain PUFAs after 12 weeks intervention in subjects with impaired glucose metabolism [17]. Fish oil supplementation was previously found to reduce the total plasma TG concentration by selectively reducing short chain fatty acids and to increase various phospholipids [27]. Thus, it is reasonable to assume that intake of fish and fish oil causes a remodeling of plasma TG species towards more long-chained fatty acids. Our results demonstrate that this remodeling occurs in healthy subjects where the total serum TG level and the BMI are unchanged.

We observed an increase in several phospholipids incorporating n-3 PUFAs, such as lysoPC(20:5) and lysoPC(22:6) in the FO group compared to the HOSO group. An association between n-3 FA intake and changes in lysoPC has previously been described [18], [28], and in accordance with our results, lysoPC(20:5) was significantly increased in subjects with impaired glucose metabolism after a healthy diet containing fatty fish [17]. In contrast, fatty fish consumption for eight weeks in subjects with CVD decreased the total concentration of lysoPC [18]. In addition, Block and colleagues found that FO supplementation increased the EPA and DHA species of lysoPC in healthy individuals [28]. The potential health effect of altering the blood plasma concentration of EPA and DHA lysoPC compounds is uncertain. However, the biological functions of lysoPC compounds are assumed to vary with the degree of saturation and acyl length [28] and LysoPC has been suggested as the major carrier of DHA to brain tissues [29].

Three out of four significantly altered SM lipids were increased in the FO group compared to the HOSO group. SM lipids are the most dominant circulating sphingolipid representing 88% of the total concentration in blood, whereasceramides account for approximately 3% [30]. SM in blood is key components and exists predominantly in the hydrophobic outer layer of lipoprotein particles. Of the lipoprotein particles, the VLDL particle contains the highest amount of SM lipids [31]. However, the localization, distribution and role SM lipid species among the lipoprotein particles is still obscure.

In the present study, FO supplementation was not associated with changes in plasma PA, lysoPE and ceramides, indicating that n-3 PUFA is selectively incorporated into other lipid classes. Ceramides have been associated with inflammation and cardiovascular disease [32], [33]. However, high content of specific C_24_ ceramides have been linked to less atherogenic lipoprotein particles in healthy subjects [31]. Lankinen et al. observed that the total concentration of ceramides decreased after fatty fish consumption for eight weeks [18]. The discrepancies observed between these studies may be due to differences in the study population and design, or due to lack of specific bioactive components in fish oil which are normally present in fish.

Lipid profiling has identified a relation between lipid acyl chain structure and risk of disease [22]. The present study shows that fish oil supplement increases the level of lipids such as TG(56:9), TG(58:10), LysoPC(22:6) and PC(38:6). These lipid species were recently associated with decreased risk of diabetes, when lipidome analyses were applied to plasma obtained from participants in the Framingham heart cohort study [22]. In that study a higher carbon number and higher double bond content were associated with decreased risk of diabetes. Thus, long-chain highly unsaturated TGs that have been associated with diabetes risk reduction were increased after intake of fish oil in the present study.

Whether the beneficial effects of fish oil supplementation may be explained by a remodeling of the plasma lipids into TGs and PLs of long-chain PUFAs, needs to be further investigated. However, PUFAs incorporated into TGs and PLs may reach tissues, cells and lipoproteins by a selective lipid exchange [34]. In the tissues, EPA and DHA can be incorporated into membranes and cause alterations in signaling pathways and the formation of lipid mediators that are important in inflammation [35], [36]. In addition, EPA and DHA or their oxidation products have the ability to activate transcription factors both in the liver and in other metabolic active tissues and increase the expression of target genes involved in lipid metabolism and inflammation [37]–[40]. Altering the lipid composition of lipoprotein particles can also contribute to modulation of the lipoprotein particles [15], including altered spatial distribution of lipids and therefore also alternation of the function [41], [42].

In conclusion, fish oil supplementation for three and seven weeks alter the plasma lipidomic profile markedly compared to intake of high-oleic sunflower oil. The selective elevation of TGs and phospholipids of high carbon number and double bond content may represent beneficial effects of fish oil supplementation in healthy subjects. Future studies are needed in order to elucidate the health benefits of incorporation of long-chain PUFAs into selective phospholipids classes and TGs.

## Supporting Information

Figure S1 **Global Root Mean Square Error plot of Y (RMSE_Y_).** RMSE of Y for the global model is plotted as a function of the number of components. Detailed explanation for the plot is given in [26].
(EPS)

Table S1 **Significantly altered lipids (Multivariate analyses, p<0.05) in the fish oil (FO) group compared to the sunflower oil (HOSO) group after three weeks intervention.** The corresponding q-values from univariate analyses are also given.
(XLS)
